# 4180 CD4 count is a prognostic marker in persons living with HIV and non-small cell lung cancer in the Bronx

**DOI:** 10.1017/cts.2020.112

**Published:** 2020-07-29

**Authors:** Madelyn Klugman, Melissa Fazzari, Mindy Ginsberg, Thomas Rohan, David Hanna, Jonathan Shuter, H. Dean Hosgood

**Affiliations:** 1 Albert Einstein College of Medicine

## Abstract

OBJECTIVES/GOALS: There is a high burden of lung cancer in persons living with HIV (PLWH). The role that HIV status, by levels of immune function and viral load, has on survival from lung cancer is not fully understood. The study’s objectives were to assess 1) the association of HIV with survival in non-small cell lung cancer (NSCLC) and 2) prognostic factors in PLWH with NSCLC. METHODS/STUDY POPULATION: Participants were from a cohort of lung cancer patients diagnosed between 2004-2017 in the Bronx, NY, with vital status ascertainment at least annually. We compared survival from NSCLC diagnosis between HIV-negative patients (HIV-, N = 2881) and PLWH (N = 88), using Cox regression, accounting for clinical and sociodemographic factors including smoking status. In three separate comparisons to HIV-, PLWH were dichotomized by CD4 count (<200 vs. ≥200 cells/μL), CD4/CD8 ratio (median, <0.43 vs. ≥0.43) and HIV viral load (VL) suppression (<75 vs. ≥75 copies/mL). In PLWH only, we assessed the relationships of CD4 count, CD4/CD8 ratio, and VL at diagnosis with survival adjusting for age, sex, and cancer stage. CD4 count and CD4/CD8 ratio were also examined as time-varying variables using a counting process approach. RESULTS/ANTICIPATED RESULTS: PLWH were younger (median 56 years, IQR 51-52 vs. 68, IQR 60-76) and more likely to be current smokers (58% vs. 37%) at diagnosis than HIV- patients. Median survival was lower in PLWH [1.1 years, 95% confidence interval (95%CI): 0.6-1.3] than in HIV- [1.6 (1.5-1.7)]. Survival comparing PLWH with higher CD4/CD8 to HIV- was similar [hazard ratio (HR), 95%CI: 0.63 (0.37-1.07)], but those with lower CD4/CD8 experienced worse survival (HR = 1.74, 95%CI: 1.07-3.89). Among PLWH, having a CD4 count < 200 cells/μL was associated with over twice the risk of death compared to those with CD4 ≥ 200 cells/μL (HR = 2.37, 95%CI: 1.14-4.92). VL and CD4/CD8 ratio were not associated with survival. Lower time-updated CD4 count was also associated with worse survival (HR = 2.19 for CD4 <200 vs. >200 cells/μL, 95%CI: 1.16-4.13). DISCUSSION/SIGNIFICANCE OF IMPACT: Among persons with NSCLC, CD4/CD8 ratio nearest diagnosis was shown to distinguish mortality risk in PLWH compared with HIV- patients. In addition, PLWH with low CD4 had worse prognosis than PLWH who had higher CD4 counts. These results suggest HIV immune status to be an essential component influencing survival in lung cancer.

